# Symptom Patterns in Adults With Cyclic Vomiting Syndrome: A 6‐Month Prospective Observational Study

**DOI:** 10.1111/nmo.14974

**Published:** 2024-12-31

**Authors:** Yaozhu J. Chen, Danielle Rodriguez, Camilla A. Richmond, William L. Hasler, David J. Levinthal, B. U. K. Li, Ioannis Petrakis, Karin S. Coyne, Melody Wu, Jan Tack, Thangam Venkatesan

**Affiliations:** ^1^ Takeda Development Center Americas Inc. Cambridge Massachusetts USA; ^2^ Evidera Bethesda Maryland USA; ^3^ Mayo Clinic Arizona Scottsdale Arizona USA; ^4^ University of Pittsburgh School of Medicine Pittsburgh Pennsylvania USA; ^5^ Medical College of Wisconsin Milwaukee Wisconsin USA; ^6^ Department of Gastroenterology University Hospital Leuven Leuven Belgium; ^7^ The Ohio State University Columbus Ohio USA

**Keywords:** cyclic vomiting syndrome, natural history, patient‐reported outcomes, retching, symptom patterns, vomiting

## Abstract

**Background:**

Data are limited on the natural history and symptom patterns of cyclic vomiting syndrome (CVS), a disorder of gut–brain interaction characterized by recurrent stereotypical vomiting, retching, and nausea episodes.

**Methods:**

A 6‐month, observational, remote study prospectively assessed symptom patterns in adults with CVS using an electronic daily diary. Patients recorded their disease experience, including CVS symptoms and associated severity, in the daily diary. The study defined a CVS episode start as the first day of 5 or more vomiting and/or retching (V + R) events. Episode end was defined as the first day of ≥ 7 (prespecified main analysis) or ≥ 4 (sensitivity analysis) days without any diary V + R events.

**Key Results:**

Eighty‐eight of 93 (94.6%) enrolled patients (62 female; mean age, 37.7 years) had daily diary data recorded during the study; 61 (69.3%) patients had at least one episode. In the prespecified main analysis, 191 episodes (median, 0.6 per 30 diary days) were reported (median duration, 3.0 days); 17.8% of the episodes lasted > 10 days. In the sensitivity analysis, 248 episodes (median, 0.7 per 30 diary days) were reported (median duration, 2.0 days). Thirteen of 88 (14.8%) patients with diary data had interepisodic V + R (reported V + R events without meeting the threshold for study‐defined episode). Other CVS‐related (non‐V + R) symptoms (most frequently nausea, abdominal pain, and sweating) were reported more frequently during the episode versus the interepisodic period.

**Conclusions and Inferences:**

This prospective longitudinal study shows marked heterogeneity of CVS episodes and highlights the need to better define and characterize episodes in these patients.

## Graphical abstract

In this prospective longitudinal study, which collected patient‐reported data on cyclic vomiting syndrome (CVS) symptom patterns daily, CVS episode number varied depending on definitions used, and 17.8% of episodes lasted > 10 days, exceeding current clinical guideline definitions. Vomiting and retching outside study‐defined episodes was reported in 14.8% of patients.

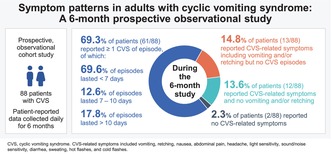

**Summary**

- In this prospective longitudinal study, which collected patient‐reported data on cyclic vomiting syndrome (CVS) symptom patterns daily, CVS episode number varied depending on definitions used; 17.8% of episodes lasted > 10 days, exceeding the episode duration defined in the Rome IV and ICHD guidelines.
- Although interepisodic vomiting and retching are not consistently recognized in the current clinical guidelines, vomiting, and retching outside study‐defined episodes were reported in 14.8% of patients from this study.
- These results highlight the need for evidence‐based episode definitions for diagnostic criteria for CVS to aid future CVS research.

## 1 Introduction

Cyclic vomiting syndrome (CVS) is a disorder of gut–brain interaction manifesting as episodes of severe vomiting and intense nausea, interspersed with periods with no or mild symptoms [1, 2, 3]. Reported prevalence is 2% in US adults [4].

CVS has four phases: prodromal/pre‐emetic, emetic, recovery, and interepisodic, and is associated with substantially diminished health‐related quality of life (HRQoL), increased health care resource utilization (HCRU), and loss of productivity [1, 4, 5, 6].

No medications are currently approved to treat CVS, but off‐label use of neuromodulators, anti‐migraine drugs, anti‐epileptics, and anti‐emetics as prophylaxis and abortive therapies is reported [7]. Additionally, patients with CVS frequently report cannabis use to alleviate nausea and vomiting [8]. Without treatment, patients may develop increased episode frequency or duration or progress to coalescent CVS, during which episodes last for weeks with almost daily symptoms [6].

CVS symptom patterns, including episode duration and time between episodes, are essential for timely CVS diagnosis and characterization. A dearth of prospective data on CVS symptom patterns led to current clinical diagnostic criteria being consensus‐driven rather than evidence‐driven [2, 7, 9, 10]. Consensus‐based definitions of episode duration vary between guidelines (< 7 days in the 2016 Rome IV guidelines [9] and ≤ 10 days in the 2016 International Classification of Headache Disorders [ICHD] guidelines [10]). The Rome IV and ICHD guideline diagnostic criterion of ≥ 1 week between episodes is also consensus‐based and requires further supporting evidence [9, 10].

We conducted a longitudinal study assessing symptom patterns of CVS, with prospective patient‐level data collected daily for 6 months. The goals were to identify meaningful clinical outcomes in CVS and to assess gaps between current clinical definitions and real‐world disease experience.

## 2 Methods

### 2.1 Study Population

Patients were sampled from the clinical database of the Medical College of Wisconsin (a tertiary CVS referral center) stored in Research Electronic Database Capture (REDCap); this database includes patients from 49 US states, ensuring geographical diversity. Patients aged ≥ 18 years were eligible if they met the Rome IV criteria for CVS (two or more vomiting episodes in the previous 6 months, each occurring ≥ 1 week apart and persisting for < 1 week; stereotypical clinical pattern; no abnormal test results accounting for vomiting) [9], as diagnosed by the principal investigator. Episode frequency had to be stable for 6 months before enrollment. Patients were excluded if they had changes to CVS prophylaxis in the 3‐month period before the screening visit or a history of alcohol or drug abuse (defined as any illicit drug use, excluding cannabis) within 6 months before the screening visit. CVS prophylaxis, abortive medications, and cannabis use were allowed and documented in the study.

The study aimed to enroll 50–100 patients with CVS; this number was selected to provide an adequate number of patients with two or more CVS episodes over the 6‐month study rather than for statistical considerations.

### 2.2 Study Design and Assessments

This prospective, observational cohort study assessing the symptom patterns of US adults with CVS was conducted between February 15, 2021, and March 7, 2022. No new treatments were initiated as part of this study. Screening and end‐of‐study visits could be completed at the study site or remotely. Patients were mailed the iPhone devices used for study monitoring upon enrollment and remotely self‐reported all daily diary assessments during the study through the study iPhone devices, with no site visits required. Each patient was followed for ≤ 6 months until the last planned visit/interaction, discontinuation from the study, or loss to follow‐up, whichever occurred first.

In this study, a remote design (enabling some or all study activities to occur at participants' homes, outside the study site) was utilized to mitigate the research challenges of CVS (highly episodic disease; most events happen at home; patients may be unable to attend study appointments during the emetic phase), minimize burden to patients' lives (no site visits required), and allow collection of continuous longitudinal data on patients' real‐world disease experience.

The study was conducted at the Medical College of Wisconsin in accordance with the ethical principles derived from the Declaration of Helsinki and enrolled patients with CVS, either residing locally or referred from other US regions. Ethics approval was provided by the Medical College of Wisconsin Institutional Review Board (approval number IRB00006380). Informed consent was obtained from all patients enrolled in this study.

### 2.3 Study Assessments

#### 2.3.1 Baseline Assessments

Demographic information was collected at the screening visit. Medical history was obtained by patient self‐report to determine conditions or diseases relevant to CVS. At the screening visit, patients were asked to report all CVS‐related symptoms ever experienced from the list of vomiting, retching, nausea, abdominal pain, headache, light sensitivity, sound/noise sensitivity, diarrhea, sweating, and hot or cold flashes; patients could also report other CVS‐related symptoms ever experienced as a free‐text entry.

#### 2.3.2 Disease Status and Changes in CVS‐Related Symptoms

During the study, the data were collected remotely via the iPhone devices (provided at the study start and returned at the study end); training on the use of study devices was provided at the screening visit. The daily time window for data entry remained constant and was based on patients' preferences recorded during the screening visit. Each day, patients were prompted to report if they had experienced any CVS‐related symptoms (individualized to CVS‐related symptom history reported during the screening visit) during the previous 24 h; no further questions were asked for that day if the patient reported “No” to the first question. Patients who answered “Yes” were provided with the list of symptoms they reported having ever experienced at the screening visit and asked to specify which symptoms they experienced that day, symptom event severity (for CVS‐related symptoms other than vomiting, retching, and diarrhea, measured on a 5‐point Likert scale from 0 [none] to 4 [very severe]), quantity (e.g., number of vomiting and/or retching [V + R] events if the patient reported V + R, or number of bowel movements if the patient reported experiencing diarrhea or increased bowel movements), and/or duration (e.g., minutes quantifying how long the patient was vomiting/retching), as applicable. Vomiting and retching were both considered in the study definition of a CVS episode because the emetic drive often persists after the stomach is completely empty during an episode, presenting as retching instead of vomiting [7].

The classification of days with diaries entry during the study is provided in Table 1. Days without diary entry during the study period were defined as missing diary days. Any days during which the patient reported any CVS‐related symptoms (including, but not limited to, V + R) were recorded as “CVS‐related symptom days.” A threshold of at least 5 reported V + R events in a day was established to define a “CVS day,” while “non‐CVS days” were defined as those with 0 to 4 V + R events; all CVS days were also CVS‐related symptom days, while non‐CVS days could be either CVS‐related symptom days or days without any CVS‐related symptoms. This threshold was based on clinical experience, aiming to differentiate the CVS emetic phase from less intense emetic gastrointestinal conditions (e.g., gastroparesis, gastroesophageal reflux). During the data analysis, episode start was defined as the first CVS day. In the prespecified main analysis, episode end was defined as the first day in a series of ≥ 7 consecutive days without V + R events, per the Rome IV criteria; missing days were not counted. To investigate whether some patients had clinical presentations with episodes occurring more closely than the 7 days, a sensitivity analysis in which episode end was defined as the first day in a series of ≥ 4 consecutive days without V + R events was performed. Episode characteristics, including episode number and duration, were based on all patients meeting the threshold for at least one episode.

**TABLE 1:** Classification of days with diary entry during the study.

Term	Definition
“CVS‐related symptom day”	Any day during which the patient reported any CVS‐related symptoms (including, but not limited to, V + R)
“CVS day”	Any day with 5 or more V + R events per day
“Non‐CVS day”	Any day with 0–4 V + R events per day

Abbreviations: CVS, cyclic vomiting syndrome; V + R, vomiting and/or retching.

Patients were also asked to record their HRQoL using the Patient‐Reported Outcomes Measurement Information System 29‐item Health Profile (PROMIS‐29) questionnaire administered weekly through the study iPhones (Data S1) [11]. Additionally, patients were asked to report HCRU (e.g., urgent care/emergency room [ER] visits or hospitalizations), interruptions to daily life (e.g., sick leave), and recent cannabis use (over the previous 24 h) in the daily diary.

#### 2.3.3 Statistical Analysis

Patient demographic and clinical characteristics, CVS‐related symptoms, HRQoL, disease burden (HCRU, interruptions to daily life), and cannabis use profiles were summarized using descriptive statistics. Outcome frequency was calculated by normalizing the number of outcome events to the number of diary days completed and reported as events per 30 diary days.

*p* values for comparison of CVS experience between male and female patients were calculated using a *t*‐test comparing group means for continuous variables and a chi‐square test for categorical variables.

## 3 Results

### 3.1 Study Population

Ninety‐three patients residing in 28 US states who were receiving their CVS care in a single tertiary CVS referral center at the Medical College of Wisconsin were screened and enrolled; 88 patients recorded daily diary data during the study and were included in the final analyses (Figure S1). Over the 6‐month study period, patients completed a median of 150.0 days (interquartile range [IQR], 91.5–169.5) of a possible 182 days of diary entry, and a median of 32.0 days (IQR, 15.0–91.0) were missing in 85 patients with missing diary days (three patients did not miss any diary days). Overall, the patients completed a median of 82.4% (IQR, 50.3–93.1) of the possible days of diary entry.

The mean ± standard deviation (SD) age of the analyzed patients was 37.7 ± 14.0 years. Most were female (70.5%) and White (84.1%) (Table 2). The median duration of living with CVS was 9.7 years (IQR, 4.8–16.0; range, 0.5–50.1). The most frequently reported comorbidities at baseline were gastroesophageal reflux disease (48.9%), anxiety (44.3%), and depression (29.5%). None of the patients were diagnosed with cannabinoid hyperemesis syndrome, though 29.5% (26/88) reported any cannabis use at baseline.

**TABLE 2:** Baseline characteristics of patients in analytical cohort.

Characteristic	Overall (*N* = 88)	Male (*n* = 26)	Female (*n* = 62)
Age, years			
Mean ± SD	37.7 ± 14.0	40.5 ± 15.7	36.6 ± 13.1
Median (IQR)	36.0 (27.0–49.0)	38.5 (28.0–51.0)	33.5 (26.0–44.0)
Race, *n* (%)			
White	74 (84.1)	24 (92.3)	50 (80.6)
Black/African American	10 (11.4)	2 (7.7)	8 (12.9)
Other	4 (4.5)	0 (0.0)	4 (6.5)
Body mass index, kg/m^2^			
Mean ± SD	28.1 ± 9.1	27.3 ± 5.9	28.4 ± 10.2
Median (IQR)	25.6 (22.0–31.3)	24.5 (23.5–29.9)	26.0 (21.3–31.7)
Smoking (nicotine) status, *n* (%)			
Current	14 (15.9)	3 (11.5)	11 (17.7)
Former (quit < 12 months ago)	1 (1.1)	1 (3.8)	0 (0.0)
Former (quit > 12 months ago)	17 (19.3)	9 (34.6)	8 (12.9)
Never	56 (63.6)	13 (50.0)	43 (69.4)
Cannabis use at baseline, *n* (%)			
Any cannabis use	26 (29.5)	9 (34.6)	17 (27.4)
Less than once a month	3 (3.4)	1 (3.8)	2 (3.2)
Once a month	1 (1.1)	0 (0.0)	1 (1.6)
A few times per month	7 (8.0)	2 (7.7)	5 (8.1)
A few times per week	8 (9.1)	4 (15.4)	4 (6.5)
Once a day	5 (5.7)	1 (3.8)	4 (6.5)
More than once per day	2 (2.3)	1 (3.8)	1 (1.6)
Time since CVS diagnosis, years			
Mean ± SD	11.5 ± 9.2	12.0 ± 10.6	11.2 ± 8.6
Median (IQR)	9.7 (4.8–16.0)	9.7 (4.7–16.9)	9.7 (5.0–15.8)
Range	0.5–50.1	1.6–50.1	0.5–40.8
Comorbidities occurring in more than one patient at screening, *n* (%)			
Gastroesophageal reflux disease	43 (48.9)	13 (50.0)	30 (48.4)
Anxiety	39 (44.3)	11 (42.3)	28 (45.2)
Depression	26 (29.5)	6 (23.1)	20 (32.3)
Migraine^a^	18 (20.5)	1 (3.8)	17 (27.4)
Hypertension	15 (17.0)	7 (26.9)	8 (12.9)
Irritable bowel syndrome	13 (14.8)	2 (7.7)	11 (17.7)
Hyperlipidemia	10 (11.4)	5 (19.2)	5 (8.1)
Asthma	7 (8.0)	3 (11.5)	4 (6.5)
Diabetes type 2	7 (8.0)	3 (11.5)	4 (6.5)
Diabetes type 1	4 (4.5)	1 (3.8)	3 (4.8)
Fibromyalgia	4 (4.5)	2 (7.7)	2 (3.2)
Bipolar disorder	3 (3.4)	1 (3.8)	2 (3.2)
Delayed gastric emptying	3 (3.4)	0 (0.0)	3 (4.8)
Obstructive sleep apnea	3 (3.4)	2 (7.7)	1 (1.6)
Posttraumatic stress disorder	3 (3.4)	0 (0.0)	3 (4.8)
Attention deficit‐hyperactivity disorder	2 (2.3)	1 (3.8)	1 (1.6)
Hypothyroidism	2 (2.3)	0 (0.0)	2 (3.2)

Abbreviations: CVS, cyclic vomiting syndrome; IQR, interquartile range; SD, standard deviation.

^a^ Includes free‐text report of Migraines, Migraines headache, Migraine headaches, Migraine headache, Migraine Headaches, Migraine, Chronic migraines.

Fifty‐three of 92 patients with available prescription data (57.6%) were receiving CVS prophylaxis (tricyclic antidepressants, 33; topiramate, 12; coenzyme Q, 12; cyproheptadine, 1 [not mutually exclusive]), with monotherapy in 39, dual therapy in 13, and triple therapy in one patient.

### 3.2 CVS Symptoms

History of nausea and V + R was reported in all patients at screening (Table 3); other most frequently reported CVS‐related symptoms at screening were history of sweating (93.2%), abdominal pain (90.9%), and cold flashes/chills (78.4%).

**TABLE 3:** Summary of CVS symptoms.

Symptom	Symptom type reported, *n* (%)		During the study					
			Number of days per patient per 30 diary days among patients who reported that specific symptom		Daily symptom severity^a^		Event quantity per day among patients who reported that specific symptom	
	History reported at screening	During the study	Mean ± SD	Median (IQR)	Mean ± SD	Median (IQR)	Mean ± SD	Median (IQR)
Vomiting and/or retching^**b**^	88 (100.0)	76 (86.4)	3.2 ± 3.4	1.8 (0.9–4.5)	NA	NA	13.6 ± 32.8	6.0 (3.0–12.0)
Nausea	88 (100.0)	85 (96.6)	7.8 ± 8.1	4.6 (1.7–10.8)	1.9 ± 0.8	2.0 (1.0–2.0)	NA	NA
Vomiting	87 (98.9)	70 (79.5)	2.3 ± 2.7	1.2 (0.6–2.9)	NA	NA	11.2 ± 31.4	5.0 (2.0–10.0)
Retching	87 (98.9)	68 (77.3)	2.4 ± 3.0	1.3 (0.4–2.9)	NA	NA	8.5 ± 16.2	4.0 (2.0–9.0)
Sweating	82 (93.2)	68 (77.3)	2.3 ± 2.7	1.6 (0.5–2.9)	2.1 ± 0.8	2.0 (2.0–3.0)	NA	NA
Abdominal pain	80 (90.9)	73 (83.0)	6.1 ± 7.4	3.1 (1.3–7.2)	2.3 ± 0.9	2.0 (2.0–3.0)	NA	NA
Cold flashes/chills	69 (78.4)	57 (64.8)	1.9 ± 2.2	1.3 (0.4–2.1)	2.0 ± 0.7	2.0 (2.0–2.0)	NA	NA
Hot flashes	65 (73.9)	53 (60.2)	1.8 ± 2.1	1.1 (0.5–2.8)	2.1 ± 0.9	2.0 (1.0–3.0)	NA	NA
Headache	64 (72.7)	58 (65.9)	5.4 ± 7.3	2.7 (1.2–5.4)	2.3 ± 0.9	2.0 (2.0–3.0)	NA	NA
Light sensitivity/phobia	59 (67.0)	45 (51.1)	4.3 ± 4.9	1.9 (0.8–4.5)	2.0 ± 0.8	2.0 (1.0–3.0)	NA	NA
Diarrhea	57 (64.8)	51 (58.0)	2.9 ± 4.8	1.2 (0.4–3.1)	NA	NA	4.5 ± 2.1	4.0 (3.0–6.0)
Sound/noise sensitivity	55 (62.5)	45 (51.1)	2.8 ± 3.4	1.7 (1.0–3.2)	2.0 ± 0.8	2.0 (2.0–2.0)	NA	NA
Other^c^	44 (50.0)	38 (43.2)	8.4 ± 12.1	3.8 (1.4–9.4)	2.1 ± 0.8	2.0 (2.0–3.0)	NA	NA

Abbreviations: CVS, cyclic vomiting syndrome; IQR, interquartile range; NA, not available; SD, standard deviation.

^a^ Symptom event severity was measured on a 5‐point Likert scale from 0 (none) to 4 (very severe).

^b^ If a patient reported vomiting and retching on the same day, the total number of vomiting and retching events was summed for that day.

^c^ Other CVS‐related symptoms reported during at least 1 day of the study were fatigue (*n =* 16), sleep issues (*n =* 12), numbness (*n =* 11), dizziness (*n =* 10), body shakes (*n =* 9), acid reflux (*n =* 7), anxiety (*n =* 7), body aches (*n =* 6), tingling in extremities (*n =* 6), back pain (*n =* 5), weakness (*n =* 5), conscious coma (*n =* 4), constipation (*n =* 4), frequent urination (*n =* 4), heart palpitations (*n =* 4), loss of appetite (*n =* 4), sensitivity to smell (*n =* 4), visual changes (*n =* 4), bloating (*n =* 3), chest pain (*n =* 3), gas (*n =* 3), heartburn (*n =* 3), hiccups (*n =* 3), lightheadedness (*n =* 3), muscle spasms (*n =* 3), rapid heartbeat (*n =* 3), vomiting (*n =* 3), burping (*n =* 2), memory issues (*n =* 2), migraine (*n =* 2), passing out (*n =* 2), restlessness (*n =* 2), tremors (*n =* 2), and weight gain (*n =* 2).

During the study, nausea was reported in 96.6% of patients (Table 3). V + R was reported in 86.4% of patients, with 77.3% reporting retching at least once during the study. Abdominal pain (83.0%), sweating (77.3%), and headache (65.9%) were the most frequently reported CVS‐related symptoms during the study other than nausea and vomiting and/or retching.

### 3.3 CVS Episodes

**FIGURE 1:**
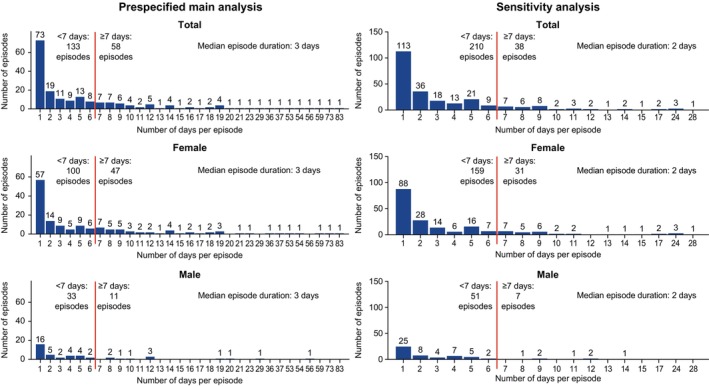
Summary of CVS episode duration in the prespecified main analysis and sensitivity analysis. The start of an episode was defined as the first CVS day (day with 5 or more V + R events). The end of an episode was defined as the first day before ≥ 7 (prespecified main analysis) or ≥ 4 (sensitivity analysis) days without any diary V + R events. Missing days were not counted in the 7 (prespecified main analysis) or 4 (sensitivity analysis) days. CVS, cyclic vomiting syndrome; V + R, vomiting and/or retching.

During the study, 61 of 88 (69.3%) patients reported at least one CVS day and were considered to have experienced a CVS episode (Table 4). In the prespecified main analysis, 191 episodes (median, 0.6 per 30 diary days; IQR, 0.4–1.1; range, 0.2–3.2) were reported (Table 4), with a median episode duration of 3.0 days (IQR, 1.0–8.0; range, 1–83). Most episodes (133/191; 69.6%) lasted < 7 days, meeting the Rome IV criteria for episode duration (Figure 1) [9]. Another 12.6% (24/191) lasted 7–10 days. The remaining 17.8% (34/191) lasted > 10 days, exceeding the CVS episode duration in the Rome IV and ICHD guidelines [9, 10]. Overall, nine of 88 (10.2%) patients met episode criteria (5 or more events in 24 h) with only retching events, while 10 (11.4%) met criteria with only vomiting events.

**TABLE 4:** Summary of patients' self‐reported CVS experience during the study.

	Overall (*N* = 88)	Male (*n* = 26)	Female (*n* = 62)	*p* (male vs. female)
Patients reporting any CVS‐related symptom days^a^, *n* (%)				
Patients meeting threshold for CVS episode (at least 1 CVS day)^b^	61 (69.3)	15 (57.7)	46 (74.2)	0.13
Patients with CVS‐related symptom days but no CVS days	25 (28.4)	10 (38.5)	15 (24.2)	0.18
Up to 4 V + R events in any CVS‐related symptom day	13 (14.8)	3 (11.5)	10 (16.1)	
CVS‐related symptom days but no V + R events	12 (13.6)	7 (26.9)	5 (8.1)	
Patients with no CVS‐related symptom days	2 (2.3)	1 (3.8)	1 (1.6)	
Number of CVS days in patients with an episode				
Patients included in the analysis, *n*	61	15	46	
Mean ± SD	8.2 ± 9.3	8.5 ± 9.0	8.2 ± 9.5	0.89
Median (IQR)	5.0 (2.0–10.0)	6.0 (4.0–10.0)	4.0 (2.0–10.0)	
Range	1.0–37.0	1.0–37.0	1.0–35.0	
Number of CVS days per 30 diary days in patients with an episode				
Patients included in the analysis, *n*	61	15	46	
Mean ± SD	2.1 ± 2.4	2.6 ± 3.5	1.9 ± 2.0	0.48
Median (IQR)	1.2 (0.4–2.9)	1.3 (0.8–2.9)	1.0 (0.4–3.1)	
Range	0.2–13.8	0.3–13.8	0.2–7.5	
Number of V + R events per day in CVS days in patients with an episode^c^				
Patients included in the analysis, *n*	61	15	46	
Mean ± SD	20.4 ± 28.0	17.9 ± 12.5	21.2 ± 31.6	0.56
Median (IQR)	12.0 (7.1–22.6)	15.8 (7.1–26.7)	11.3 (7.0–20.2)	
Range	5.0–184.4	5.0–46.4	5.0–184.4	
Number of vomiting events per day in CVS days^d^				
Patients included in the analysis, *n*	55	14	41	
Mean ± SD	14.3 ± 24.1	11.0 ± 9.0	15.4 ± 27.4	0.44
Median (IQR)	8.0 (5.0–14.9)	7.6 (4.0–16.0)	8.3 (5.8–14.3)	
Range	1.5–167.8	1.5–33.1	2.0–167.8	
Number of retching events per day in CVS days^e^				
Patients included in the analysis, *n*	57	14	43	
Mean ± SD	12.0 ± 13.4	10.7 ± 9.3	12.4 ± 14.5	0.69
Median (IQR)	6.5 (4.8–13.5)	6.3 (4.8–17.8)	7.7 (4.0–13.5)	
Range	1.0–75.0	1.0–29.0	2.0–75.0	
Number of CVS‐related symptom days in patients with no CVS days				
Patients included in the analysis, *n*	25	10	15	
Mean ± SD	15.2 ± 27.5	17.6 ± 32.9	13.7 ± 24.4	0.73
Median (IQR)	6.0 (2.0–11.0)	4.5 (1.0–16.0)	7.0 (2.0–10.0)	
Range	1.0–108.0	1.0–108.0	1.0–99.0	
Number of CVS‐related symptom days per 30 diary days in patients with no CVS days				
Patients included in the analysis, *n*	25	10	15	
Mean ± SD	8.0 ± 9.9	9.7 ± 11.8	6.9 ± 8.6	0.51
Median (IQR)	3.4 (1.4–12.0)	3.9 (1.4–17.1)	3.4 (1.1–12.0)	
Range	0.2–30.0	0.2–30.0	0.2–28.6	

Abbreviations: CVS, cyclic vomiting syndrome; IQR, interquartile range; SD, standard deviation; V + R, vomiting and/or retching.

^a^ CVS‐related symptom day was defined as any day during which the patient self‐reported experiencing any CVS‐related symptoms (including, but not limited to, V + R) over the past 24 h.

^b^ CVS day was defined as any day with 5 or more V + R events/day.

^c^ If a patient reported vomiting and retching on the same day, the numbers of vomiting and retching events were summed for that day.

^d^ In patients who reported vomiting on any CVS day.

^e^ In patients who reported retching on any CVS day.

Twenty‐five (28.4%) patients reported only interepisodic symptoms (CVS‐related symptom days but no CVS days/episodes); of these, 13 reported interepisodic V + R. The 13 patients with interepisodic V + R but no CVS days reported 33 days with V + R overall, for a median of 1 day per patient (IQR, 1–4; range, 1–8). On these days, the patients experienced V + R for a median of two times per day (IQR, 1–3). Only two patients (2.3%) reported no CVS‐related symptom days throughout the 6‐month study.

To investigate if some of the 17.8% of episodes exceeding guideline‐defined duration may have instead comprised two or more distinct episodes with intervals of V + R–free days shorter than the guideline criterion of 7 days, we performed a sensitivity analysis with a threshold of ≥ 4 consecutive days without V + R events between episodes. In this analysis, the number of episodes increased from 191 to 248 (median, 0.7 per 30 diary days; IQR, 0.4–1.3; range, 0.2–3.2) (Table 5). Episodes lasted for a median of 2.0 days (IQR, 1.0–5.0; range, 1–28). Most episodes (210/248; 84.7%) lasted < 7 days, 9.3% (23/248) lasted 7–10 days, and only 6.0% (15/248) lasted for > 10 days in this sensitivity analysis.

**TABLE 5:** Summary of CVS episodes in the prespecified main analysis and sensitivity analysis.

	Prespecified main analysis (≥ 7 days between episodes)				Sensitivity analysis (≥ 4 days between episodes)			
	Overall (*N* = 88)	Male (*n =* 26)	Female (*n =* 62)	*p* (male vs. female)	Overall (*N* = 88)	Male (*n =* 26)	Female (*n =* 62)	*p* (male vs. female)
Patients included in the analysis, *n*	61	15	46		61	15	46	
Number of episodes^a^	191	44	147		248	58	190	
Mean ± SD	3.1 ± 2.2	2.9 ± 1.6	3.2 ± 2.4	0.22	4.1 ± 3.4	3.9 ± 3.7	4.1 ± 3.4	0.30
Median (IQR)	2.0 (1.0–4.0)	3.0 (1.0–5.0)	2.0 (1.0–4.0)		3.0 (1.0–6.0)	3.2 (1.0–5.0)	3.5 (1.0–6.0)	
Range	1.0–12.0	1.0–5.0	1.0–12.0		1.0–15.0	1.0–15.0	1.0–14.0	
Number of episodes per 30 diary days								
Mean ± SD	0.8 ± 0.6	0.8 ± 0.5	0.8 ± 0.6	0.40	1.0 ± 0.7	1.0 ± 0.8	1.0 ± 0.7	0.38
Median (IQR)	0.6 (0.4–1.1)	0.8 (0.4–1.0)	0.6 (0.4–1.1)		0.7 (0.4–1.3)	0.8 (0.4–1.2)	0.7 (0.4–1.3)	
Range	0.2–3.2	0.2–2.3	0.2–3.2		0.2–3.2	0.2–2.6	0.2–3.2	
Episode duration, days								
Mean ± SD	7.1 ± 12.0	6.3 ± 9.7	7.4 ± 12.6	0.52	3.6 ± 4.2	3.3 ± 3.2	3.6 ± 4.5	0.55
Median (IQR)	3.0 (1.0–8.0)	3.0 (1.0–7.0)	3.0 (1.0–8.0)		2.0 (1.0–5.0)	2.0 (1.0–4.0)	2.0 (1.0–5.0)	
Range	1.0–83.0	1.0–56.0	1.0–83.0		1.0–28.0	1.0–14.0	1.0–28.0	
Cumulative duration of episodes per patient, days								
Mean ± SD	22.3 ± 30.4	18.3 ± 26.0	23.7 ± 31.9	0.28	14.5 ± 19.0	12.9 ± 13.5	15.1 ± 20.5	0.27
Median (IQR)	12.0 (3.0–24.0)	12.0 (7.0–16.0)	12.0 (2.0–32.0)		8.0 (3.0–16.0)	9.0 (7.0–15.0)	5.5 (2.0–21.0)	
Range	1.0–142.0	1.0–107.0	1.0–142.0		1.0–95.0	1.0–58.0	1.0–95.0	

Abbreviations: CVS, cyclic vomiting syndrome; IQR, interquartile range; SD, standard deviation; V + R, vomiting and/or retching.

^a^ The start of an episode was defined as the first CVS day (day with 5 or more V + R events). The end of an episode was defined as the first day before ≥ 7 (prespecified main analysis) or ≥ 4 (sensitivity analysis) days without any diary V + R events. Missing days were not counted in the 7 (prespecified main analysis) or 4 (sensitivity analysis) days.

**FIGURE 2:**
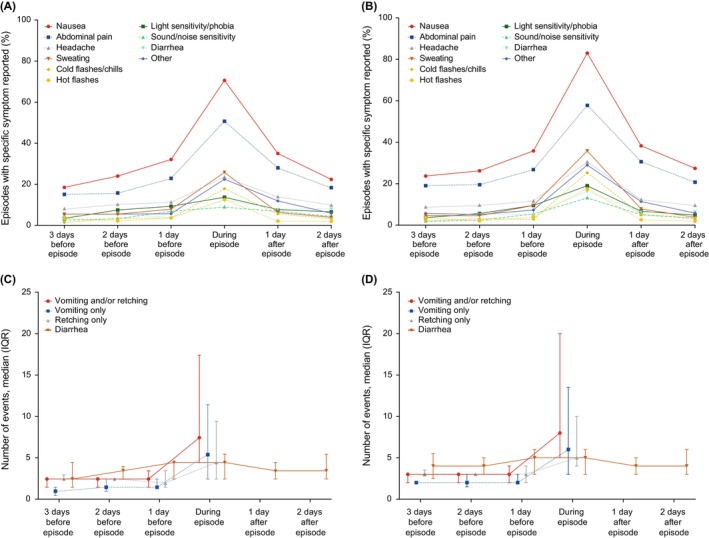
CVS‐related symptoms before, during, and after the CVS episode in the (A, C) prespecified main analysis and (B, D) sensitivity analysis. The start of an episode was defined as the first CVS day (day with 5 or more V + R events). The end of an episode was defined as the first day before ≥ 7 (prespecified main analysis) or ≥ 4 (sensitivity analysis) days without any diary V + R events. Missing days were not counted in the 7 (prespecified main analysis) or 4 (sensitivity analysis) days. CVS, cyclic vomiting syndrome; IQR, interquartile range; V + R, vomiting and/or retching.

The 61 patients with an episode reported a median of 5.0 CVS days (IQR, 2.0–10.0; range, 1.0–37.0) over the study period (median, 1.2 per 30 diary days [IQR, 0.4–2.9; range, 0.2–13.8]). During CVS days, a median of 12.0 V + R events per day (IQR, 7.1–22.6; range, 5.0–184.4) were reported. Overall, 55 patients reported vomiting (median, 8.0 events per day [IQR, 5.0–14.9; range, 1.5–167.8]) and 57 reported retching (median, 6.5 events per day [IQR, 4.8–13.5; range, 1.0–75.0]) during CVS days (Table 4). There were no significant differences in patient‐reported CVS experience between male and female patients (Tables 4 and 5). CVS‐related symptoms other than V + R were also reported more frequently during episodes versus the interepisodic period (Figure 2).

### 3.4 HRQoL

During the closest week before an episode, the mean ± SD of PROMIS‐29 Summary scales in patients with CVS were 44.5 ± 9.9 for Physical Health and 43.6 ± 8.9 for Mental Health. Both were lower than the general population (mean ± SD, 50.0 ± 10.0) [11], suggesting clinically significant (5‐point change in PROMIS score [12]) impairment of HRQoL, even during the interepisodic phase. During a week with an episode, the PROMIS‐29 summary scores were reduced further (mean ± SD, 39.1 ± 9.1 and 39.6 ± 7.1 for Physical and Mental Health Summary scales, respectively).

### 3.5 HCRU And Interruptions to Regular Life

A higher proportion of patients reported medical care on CVS versus non‐CVS days (Table 6). Of the 61 patients with an episode, more than a third (21 patients) reported at least one ER visit during the study; 71.4% of these reported more than one ER visit. Thirteen patients (21.3%) reported at least one hospital admission. Additionally, 93.4% of patients with an episode reported inability to complete daily activities (mean ± SD, 4.7 ± 6.3 days per 30 diary days), 50.8% reported missing work (2.0 ± 2.4 days per 30 diary days), and 16.4% reported missing school (1.3 ± 1.6 days per 30 diary days) (Table 6).

**TABLE 6:** Summary of self‐reported HCRU and interruptions to daily life owing to CVS in patients with an episode.

	Overall (*n* = 61)	During CVS days^a^ (*n =* 61)	During non‐CVS days^b^ (*n =* 61)
Self‐reported HCRU			
Emergency room visits			
Patients reporting, *n* (%)	21 (34.4)	19 (31.1)	9 (14.8)
Frequency of visits, mean ± SD	5.1 ± 7.6	4.5 ± 7.6	2.2 ± 2.4
Hospital admission/inpatient care			
Patients reporting, *n* (%)	13 (21.3)	11 (18.0)	8 (13.1)
Frequency of visits, mean ± SD	4.9 ± 6.0	2.3 ± 1.3	4.4 ± 5.4
Gastroenterologist visit			
Patients reporting, *n* (%)	5 (8.2)	4 (6.6)	1 (1.6)
Frequency of visits, mean ± SD	3.2 ± 2.3	3.5 ± 2.5	1.5 ± 0.7
Urgent care			
Patients reporting, *n* (%)	6 (9.8)	6 (9.8)	1 (1.6)
Frequency of visits, mean ± SD	1.2 ± 0.4	1.0 ± 0.0	1.0 ± 0.0
Primary care doctor			
Patients reporting, *n* (%)	4 (6.6)	3 (4.9)	2 (3.3)
Frequency of visits, mean ± SD	1.3 ± 0.5	1.0 ± 0.0	1.0 ± 0.0
Other			
Patients reporting, *n* (%)	20 (32.8)	13 (21.3)	14 (23.0)
Frequency of visits, mean ± SD	9.3 ± 19.9	2.9 ± 4.4	9.9 ± 21.6
Interruptions to regular life			
Any interruption to regular life			
Have symptom interruptions, *n* (%)	60 (98.4)	58 (95.1)	57 (93.4)
Number of days with interruptions per 30 diary days, mean ± SD	5.5 ± 6.4		
Percentage of days with interruptions per person^c^, %, mean ± SD		90.6 ± 18.9	15.2 ± 21.4
Inability to complete daily activities			
Have symptom interruptions, *n* (%)	57 (93.4)	51 (83.6)	51 (83.6)
Number of days with interruptions per 30 diary days, mean ± SD	4.7 ± 6.3		
Percentage of days with interruptions per person^c^, %, mean ± SD		75.5 ± 30.9	14.0 ± 21.7
Missed work			
Have symptom interruptions, *n* (%)	31 (50.8)	26 (42.6)	28 (45.9)
Number of days with interruptions per 30 diary days, mean ± SD	2.0 ± 2.4		
Percentage of days with interruptions per person^c^, %, mean ± SD		59.0 ± 31.1	4.1 ± 5.2
Missed school			
Have symptom interruptions, *n* (%)	10 (16.4)	8 (13.1)	6 (9.8)
Number of days with interruptions per 30 diary days, mean ± SD	1.3 ± 1.6		
Percentage of days with interruptions per person^c^, %, mean ± SD		45.5 ± 29.3	3.5 ± 2.7
Other			
Have symptom interruptions, *n* (%)	44 (72.1)	31 (50.8)	34 (55.7)
Number of days with interruptions per 30 diary days, mean ± SD	1.2 ± 1.2		
Percentage of days with interruptions per person^c^, %, mean ± SD		44.9 ± 34.8	3.6 ± 3.8

Abbreviations: CVS, cyclic vomiting syndrome; HCRU, health care resource utilization; SD, standard deviation; V + R, vomiting and/or retching.

^a^ CVS day was defined as any day with 5 or more V + R events/day.

^b^ Non‐CVS day was defined as any day with 0 to 4 V + R events/day.

^c^ Number of days with interruptions divided by the number of CVS/non‐CVS days for each participant among participants with interruptions.

Medications used by the patients during the study were reported as free‐text entries in the “other HCRU” section (Table S1).

### 3.6 Cannabis Use

Of the 26 (29.5%) patients who reported baseline cannabis use, 25 also reported cannabis use during the study. Eighteen of the 25 (72.0%) on‐study cannabis users experienced an episode. Among these 18 patients, the percentage of diary days with cannabis use normalized across the total number of diary days each patient completed was 60.2% ± 34.0 (mean ± SD), with a mean ± SD 3.2 ± 2.4 times of cannabis use per day on days with reported use.

Among the 18 cannabis users with an episode, the percentages of days with cannabis use were similarly distributed between CVS and non‐CVS days (mean ± SD, 60.2% ± 40.9 vs. 60.0% ± 35.2, respectively); 4 cannabis users reported use only during non‐CVS days. Cannabis use frequency per day was numerically higher on CVS versus non‐CVS days (mean ± SD, 3.8 ± 2.6 vs. 3.2 ± 2.5 times per day, respectively).

## 4 Discussion

To our knowledge, this is the first prospective longitudinal study collecting daily patient‐level data in CVS via a remote design. The study population was predominantly female and White, in line with several retrospective analyses and case series in adults with CVS [4, 5, 6, 13].

We used a combination of V + R events to define episode start and end. Because attempts to vomit when the stomach is empty may present as retching, V + R events combined may better define episodes and their burden versus vomiting events alone. On CVS days, patients reported a median of 8.0 vomiting and 6.5 retching events, for a median of 12.0 V + R events combined, suggesting that vomiting and retching frequently occur together and independently contribute to episode burden. The importance of retching as an additional burden, independent from vomiting, may be underrecognized in current clinical practice [7, 9, 10].

Study results highlight that the episode number depends on definitions used. In the prespecified analysis using the Rome IV guideline threshold of ≥ 7 days without V + R events between episodes, 191 episodes (1–12 per patient; 0.2–3.2 per 30 diary days; duration, 1–83 days) were reported. Only 69.6% of episodes met the Rome IV diagnostic criterion of < 7 days' duration [9]; 82.2% of episodes met the ICHD diagnostic criterion of ≤ 10 days' duration, including 12.6% of episodes lasting 7–10 days [10]. The remaining 17.8% of episodes lasted > 10 days and exceeded all current diagnostic criteria for CVS episode duration, suggesting that the definition of < 7 days' duration may be too restrictive and lead to underdiagnosis of CVS or misclassify a large subset of patients as having coalescent CVS or chronic nausea vomiting syndrome. However, the patients enrolled in the study had a median of 9.7 years after CVS diagnosis and were enrolled through a tertiary CVS referral center, thus potentially having more severe disease; episodes in these patients may have been longer versus those newly diagnosed with CVS, and some long episodes may have been due to true coalescent CVS.

Using a sensitivity analysis (threshold of ≥ 4 days without V + R events between episodes), the number of episodes increased from 191 to 248, and the maximum reported duration of an episode decreased from 83 to 28 days. These heterogeneous and skewed data suggest that restrictive, universal definitions requiring ≥ 7 days without V + R between episodes may not adequately reflect clinical reality and may lead to underdiagnosis of CVS and delays in timely care. Allowing shorter time intervals between episodes or using a notable reduction from the peak V + R frequency versus a complete absence of V + R to define episode end may need to be considered to align with the clinical reality. Care should be taken to retain the distinction between CVS and chronic vomiting, avoiding misdiagnosing patients with coalescent CVS who may have < 7 days without V + R between episodes.

Consistent with the literature, CVS episodes were associated with a high frequency of other CVS‐related symptoms in addition to V + R. However, 28.4% of patients reported CVS‐related symptoms, including vomiting, outside the predefined study threshold for an episode. Interepisodic V + R without any study‐defined episodes was observed in 14.8% of patients, potentially driven by abortive medications modifying an episode. These data suggest that CVS‐related symptoms outside episodes, including in the prodromal phase, are relatively common in CVS. Interepisodic symptoms are acknowledged in the Rome IV diagnostic criteria, whereas the ICHD definition of CVS describes complete freedom from symptoms between attacks [9, 10].

With no evidence‐based definition in current guidelines, we used an empirical threshold of five or more V + R events in a day to define episode start. In the clinical setting, some patients with CVS may start an episode with prodromal symptoms but have fewer than 5 V + R events, not meeting the study‐defined threshold for an episode [14]. Taken together, this highlights the need for evidence‐based guideline definitions of episode (emetic phase) beginning and end and the inclusion of other CVS‐related symptoms during the interepisodic phase.

Episodes were associated with reduced HRQoL. Daily life disruptions (e.g., missed days of school/work) and CVS‐related medical care were reported on and outside CVS days, and a higher proportion of patients with an episode during the study reported HCRU on CVS days; almost one‐quarter (21.3%) of patients reported at least one hospitalization. HRQoL was impaired even during the interepisodic phase, highlighting that patients with CVS remain affected even when they are “well.” These data call for a more holistic evaluation of disease burden beyond number of episodes or V + R days.

Almost one‐third of patients reported cannabis use during the study, consistent with previous studies reporting cannabis use among US adults with CVS [4, 15, 16, 17]. The 18 patients with an episode and any cannabis use reported using cannabis on approximately 60% of diary days on average. Although the study collected data on frequency of cannabis use per day, it did not collect data on dosage or mode of administration at each use. Furthermore, cannabis users may have chosen not to report cannabis use at screening, and only those who reported use at screening were subsequently asked about cannabis use in the daily diary. During the study, some patients reported using cannabis more than three times in a day, raising the possibility of undiagnosed cannabinoid hyperemesis syndrome. Although the study results do not allow us to clarify whether cannabis may have triggered or alleviated CVS symptoms, relief of CVS symptoms with cannabis use was reported previously [17]. Larger prospective studies are warranted to better understand the relationship between cannabis use, hyperemesis, and CVS [8].

This study was conducted in patients from 28 US states treated in one referral center for CVS, which may have contributed to referral bias in patient selection; furthermore, the study results may not representatively reflect the experience of patients with CVS globally. Although no treatments are currently approved by the US Food and Drug Administration for CVS management, prophylactic and abortive therapies are nevertheless used in clinical practice based on expert opinion and clinical experience. Because it would be unethical to withhold therapies in a noninterventional study, these existing treatments (e.g., ondansetron and triptans) were allowed during the study period and could have altered the disease experience. Nevertheless, it has been reported that most patients with CVS receiving current care continue to experience episodes [18]. In our study, episodes were reported in 69.3% of patients, and V + R without meeting the episode threshold was reported in an additional 14.8%, despite a potential confounder of CVS prophylaxis, which was used by > 50% of enrolled patients with available data.

Despite these limitations, this study contributes to a deeper understanding of disease experience and symptom patterns in patients with CVS over time. The results emphasize the high clinical, humanistic, and economic burden of CVS and call for revision of current consensus‐based diagnostic criteria, as well as updated guidelines to support diagnosis, management, and clinical development. Future guidelines should ideally be harmonized and provide evidence‐based definitions of CVS episodes (including thresholds for episode start and end) and interepisodic disease burden.

## 5 Conclusions

To conclude, in this prospective longitudinal study collecting daily, self‐reported patient‐level data, patients with CVS reported high variation in the number and duration of CVS episodes, highlighting the unmet need for evidence‐based guideline definitions for episode start and end, and the frequent presence of CVS symptoms during the interepisodic phase. These data elucidate the symptom patterns of CVS and may contribute to informing the revised diagnostic criteria for CVS.

## Author Contributions

**Yaozhu J. Chen:** planning and/or conducting the study; collecting and/or interpreting the data; drafting the manuscript; approved the final draft submitted. **Danielle Rodriguez:** collecting and/or interpreting the data; drafting the manuscript; approved the final draft submitted. **Camilla A. Richmond:** planning and/or conducting the study; collecting and/or interpreting the data; drafting the manuscript; approved the final draft submitted. **William L. Hasler:** collecting and/or interpreting the data; drafting the manuscript; approved the final draft submitted. **David J. Levinthal:** drafting the manuscript; approved the final draft submitted. **B. U. K. Li:** drafting the manuscript; approved the final draft submitted. **Ioannis Petrakis:** planning and/or conducting the study; collecting and/or interpreting the data; drafting the manuscript; approved the final draft submitted. **Karin S. Coyne:** collecting and/or interpreting the data; drafting the manuscript; approved the final draft submitted. **Jan Tack:** drafting the manuscript; approved the final draft submitted. **Thangam Venkatesan:** planning and/or conducting the study; collecting and/or interpreting the data; drafting the manuscript, approved the final draft submitted. All authors approved the final version of the manuscript.

## Ethics Statement

This study was conducted with the approval of the Medical College of Wisconsin Institutional Review Board (approval number IRB00006380) in accordance with the ethical principles derived from the Declaration of Helsinki, the International Conference of Harmonization Good Clinical Practice: Consolidated Guideline, and all applicable laws and regulations. Informed consent was obtained from all patients enrolled in this study.

## Conflicts of Interest

Yaozhu J. Chen and Camilla A. Richmond were employees of Takeda at the time the study was conducted and the manuscript developed. Danielle Rodriguez is an employee of Evidera, which was contracted by Takeda for work relating to this study. Ioannis Petrakis and Melody Wu are employees of the study sponsor, Takeda, and hold Takeda stock or stock options. William L. Hasler reports advisory board participation for Enterra Medical and is an author and a reviewer for UpToDate. David J. Levinthal reports fees from Aditum Bio, Alexza Pharmaceuticals, Mahana, and Takeda. B.U.K. Li reports consultancy fees from Takeda and is a section editor for UpToDate. Karin S. Coyne is an employee of Evidera, which was contracted by Takeda for work relating to this study, and is a scientific consultant for Takeda. Jan Tack reports consultancy fees and grant support from Takeda. Thangam Venkatesan reports consultancy fees and grant support for education from Takeda. Medical writing support was provided by Milda Tyler, PhD, of Excel Scientific Solutions Inc., and funded by Takeda.

## Supporting information


Data S1.

## Data Availability

The datasets, including the redacted study protocol, redacted statistical analysis plan, and individual participants' data supporting the results reported in this article, will be made available within 3 months from the initial request to researchers who provide a methodologically sound proposal. The data will be provided after its deidentification, in compliance with applicable privacy laws, data protection, and requirements for consent and anonymization.
