# Supplementary material for: Symptom Patterns in Adults With Cyclic Vomiting Syndrome: A 6‐Month Prospective Observational Study
Source: Neurogastroenterol Motil. 2024 Dec 31;37(2):e14974. doi: 10.1111/nmo.14974 (PMC11748820; doi:10.1111/nmo.14974)
Supplement: Supplementary file 1 — Data S1. [file NMO-37-e14974-s001.docx]

SUPPORTING INFORMATION

Supplementary methods

**Health-related quality-of-life measurements using PROMIS-29**

The Patient-Reported Outcomes Measurement Information System 29-item Health Profile (PROMIS-29) questionnaire measures health-related quality of life over seven domains (physical functioning, fatigue, pain interference, depressive symptoms, anxiety, ability to participate in social roles and activities, and sleep disturbance); and pain intensity using a single-item 11-point rating scale.^1,2^ Each of the seven domains is measured over four items; each item uses a five-category response scale and a 7-day recall period, except for the physical functioning and ability to participate in social roles and activities domains, which do not have a recall period. The scores are then rescaled to a standardized T-score, where the general population T-score is set to 50, with a standard deviation of 10.^2^ The PROMIS-29 Physical Health Summary Score is calculated from physical function, ability to participate in social roles and activities, pain (interference and intensity), and fatigue domain scores, and the PROMIS-29 Mental Health Summary Score is calculated from fatigue, anxiety and depressive symptoms, sleep disturbance, ability to participate in social roles and activities, and pain (interference and intensity) domain scores.^1^ A five-point difference in a PROMIS-29 measure score is considered to be a clinically significant difference.^3^

Supplementary references

1. Hays RD, Spritzer KL, Schalet BD, Cella D. PROMIS^®^-29 v2.0 profile physical and mental health summary scores. *Qual Life Res.* 2018;27(7):1885-1891. doi:10.1007/s11136-018-1842-3.

2. Cella D, Choi SW, Condon DM, et al. PROMIS^®^ adult health profiles: efficient short-form measures of seven health domains. *Value Health.* 2019;22(5):537-544. doi:10.1016/j.jval.2019.02.004.

3. LaVela SL, Etingen B, Miskevics S, Cella D. Use of PROMIS-29® in US veterans: diagnostic concordance and domain comparisons with the general population. *J Gen Intern Med.* 2019;34(8):1452-1458. doi:10.1007/s11606-019-05011-9.

**FIGURE S1** Study compliance of patients.


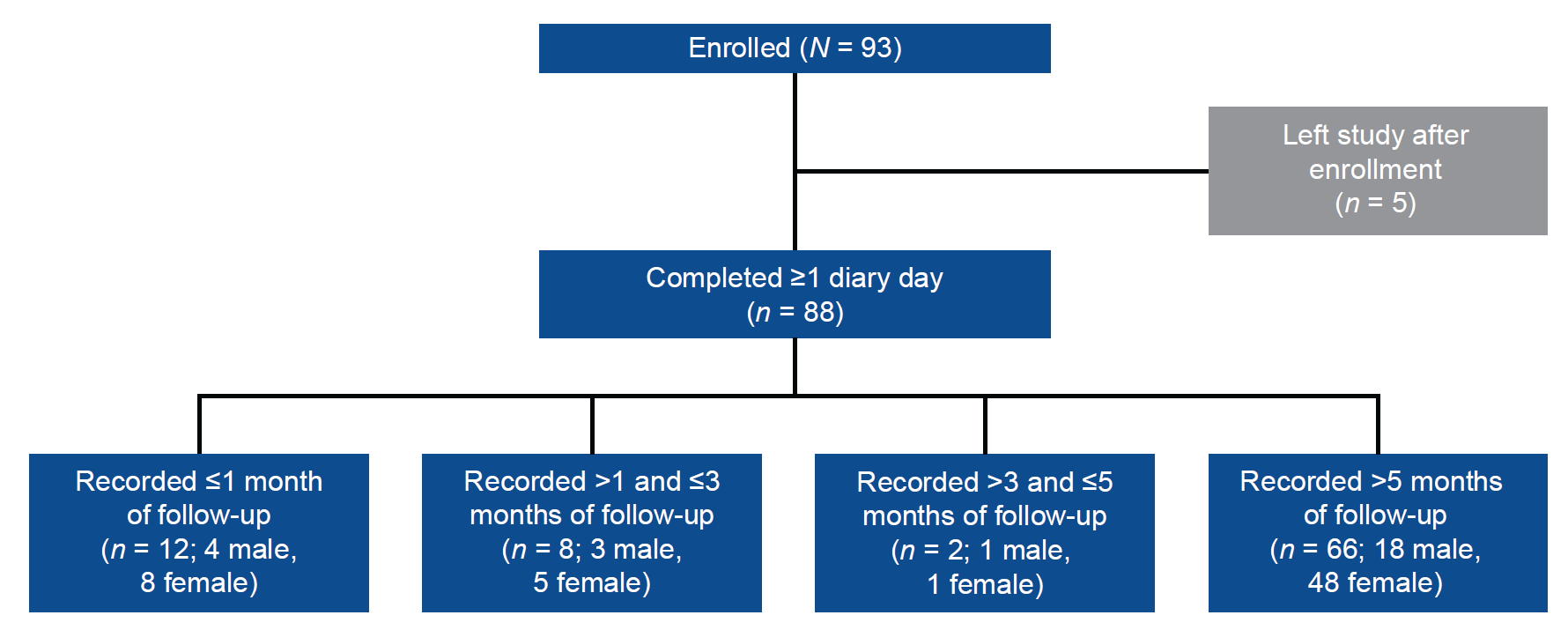


**TABLE S1** Descriptive summary of self-reported use of CVS medications.

| **Self-reported entry** | **Generic name^†^** | **Number of days reported** |
| --- | --- | --- |
| “Benadryl” | Diphenhydramine | 35 |
| “home infusion of IV Zofran” | Ondansetron | 17 |
| “fluids” |  | 15 |
| “Zofran” | Ondansetron | 11 |
| “morphine” |  | 11 |
| “promethazine” |  | 10 |
| “Phenergan suppositories” | Promethazine | 8 |
| “Tylenol” | Acetaminophen | 7 |
| "home infusion” |  | 5 |
| “Emend” | Aprepitant | 4 |
| “IV pain medication” |  | 3 |
| “anti-diarrhea” |  | 2 |
| “anti-nausea medication” |  | 2 |
| “sumatriptan” |  | 2 |
| “aprepitant” |  | 1 |
| “cannabis” |  | 1 |
| “Dilaudid” | Hydromorphone | 1 |
| “Excedrin” | Acetaminophen, aspirin, and caffeine | 1 |
| “ibuprofen” |  | 1 |
| “IV fluids” |  | 1 |
| “lidocaine” |  | 1 |
| “lorazepam” |  | 1 |
| “Midol” | Acetaminophen, caffeine, and pyrilamine | 1 |

*Note:* The reason for the use of medications (i.e., prophylaxis or abortion of an episode) was not reported.

^†^Where applicable.

Abbreviations: CVS, cyclic vomiting syndrome; IV, intravenous.
